# Gene Expression Changes of Murine Cortex Homeostasis in Response to Sleep Deprivation Hint Dysregulated Aging-like Transcriptional Responses

**DOI:** 10.3390/brainsci12070825

**Published:** 2022-06-24

**Authors:** Panagiotis Giannos, Konstantinos Prokopidis, Scott C. Forbes, Kamil Celoch, Darren G. Candow, Jaime L. Tartar

**Affiliations:** 1 Department of Life Sciences, Faculty of Natural Sciences, Imperial College London, South Kensington, London SW7 2AZ, UK; 2 Society of Meta-Research and Biomedical Innovation, London W12 0BZ, UK; k.prokopidis@liverpool.ac.uk; 3 Department of Musculoskeletal Biology, Institute of Life Course and Medical Sciences, University of Liverpool, Liverpool L69 3BX, UK; 4 Department of Physical Education Studies, Faculty of Education, Brandon University, Brandon, MB R7A 6A9, Canada; forbess@brandonu.ca; 5 Department of Psychology and Neuroscience, Nova Southeastern University, Fort Lauderdale, FL 33314, USA; kceloch@nova.edu (K.C.); tartar@nova.edu (J.L.T.); 6 Faculty of Kinesiology and Health Studies, University of Regina, Regina, SK S4S 0A2, Canada; darren.candow@uregina.ca

**Keywords:** aging, cerebral cortex, sleep deprivation, homeostasis, differentially expressed genes

## Abstract

Sleep deprivation leads to the deterioration in the physiological functioning of the brain, cognitive decline, and many neurodegenerative diseases, all of which progress with advancing age. Sleep insufficiency and impairments in cognitive function are characterized by progressive neuronal losses in the cerebral cortex. In this study, we analyze gene expression profiles following sleep-deprived murine models and circadian matched controls to identify genes that might underlie cortical homeostasis in response to sleep deprivation. Screening of the literature resulted in three murine (*Mus musculus*) gene expression datasets (GSE6514, GSE78215, and GSE33491) that included cortical tissue biopsies from mice that are sleep deprived for 6 h (*n* = 15) and from circadian controls that are left undisturbed (*n* = 15). Cortical differentially expressed genes are used to construct a network of encoded proteins that are ranked based on their interactome according to 11 topological algorithms. The analysis revealed three genes—NFKBIA, EZR, and SGK1—which exhibited the highest multi-algorithmic topological significance. These genes are strong markers of increased brain inflammation, cytoskeletal aberrations, and glucocorticoid resistance, changes that imply aging-like transcriptional responses during sleep deprivation in the murine cortex. Their potential role as candidate markers of local homeostatic response to sleep loss in the murine cortex warrants further experimental validation.

## 1. Introduction

Sleep deprivation is an established global public health burden which is associated with cognitive impairments, including reduced psychomotor speed, attention, perception, and overall memory [1,2,3,4,5].

Pathophysiologically, declines in sleep sufficiency and cognitive function are characterized by progressive neuronal losses in the cortex via β-amyloid peptides and intracellular neurofibrillary tangles from tau protein hyperstimulation [6]. Recent in vivo findings have highlighted the potential of sleep deprivation in generating tauopathy and gliosis that are linked to increased stress responses through disrupting the cortical and thalamic synaptic proteome [7,8]. During sleep deprivation, these alterations may mediate protein synthesis along with prefrontal functional and neuronal connectivity changes in the cerebral cortex [9,10], along with the elimination of dendritic spines [11]. Specifically, sleep deprivation may attenuate the mammalian target of rapamycin complex 1 phosphorylation and mRNA translation in the cortex [12,13], explaining further the cognitive-induced impairments from sleep deprivation. Accordingly, the pervasion of sleep disruption-induced cognitive impairment may accelerate abnormal brain aging, contributing to perturbed hippocampal and prefrontal-dependent learning ability and memory function [14,15].

Previous studies identifying causal genes, networks, and transcriptional regulators have revealed multiple candidates as mediators of sleep deprivation on interaction components of the hypothalamus [16] and the hippocampus [17]. However, evidence of gene expression alterations related to cortical changes following sleep deprivation remains scarce. Nevertheless, the cortex is particularly vulnerable to the effects of sleep deprivation and the disruption of circadian rhythmicity [18]. Likewise, cortical atrophy has been longitudinally correlated with sleep quality in community-dwelling populations [19]. Therefore, efforts to identify key regulatory genes linked with sleep deprivation may provide insights into possible underlying molecular mechanisms that are associated with age-related cortical pathway senescence. Additionally, the exploration of key regulatory genes can assist with the development of potential treatments against cortical burden in response to sleep insufficiency. To this end, we compared gene expression profiles in the cerebral cortex of sleep-deprived murine models with circadian-matched controls. Our aim was to identify differentially expressed genes (DEGs) whose dysregulated expression and protein interactome were linked with sleep deprivation in the murine cortex.

## 2. Methods

### 2.1. Collection of Microarray Datasets

The literature was screened from inception until January 2022 by searching the National Center for Biotechnology Information (NCBI) GEO using the following search terms: (sleep AND deprivation OR restriction OR loss) and (brain OR cortex). A further search was performed through the National Library of Medicine (NLM) PubMed using the following additional terms: (differentially expressed genes OR DEGs). Two authors (PG and KP) created the search strategy and conducted the screening of the retrieved datasets.

Datasets were restricted based on organism type (*Mus musculus*), expression profiling (microarray), sample type (cerebral cortex), and condition (sleep deprivation). No search restrictions were employed and datasets in absence of expression data for controls were omitted. No further exclusion criteria based on the baseline characteristics of murine models, from which cortical tissue samples were retrieved, were considered.

### 2.2. Identification of Differentially Expressed Genes

Cerebral cortex samples from mice that were sleep deprived were compared to circadian-matched controls that were left undisturbed. DEGs were obtained through the random effect model that was ensued for the integration of differential gene expression using ImaGEO [20]. Genes with the strongest average effect between all included datasets were retrieved [21]. Significant DEGs were defined based on a *p* < 0.05 corrected by the Benjamini-Hochberg False Discovery Rate and those with a *Z*-score > 1.96 were considered as upregulated, while those with a *Z*-score < 1.96 as downregulated (both corresponding to a 5% significance level).

### 2.3. Construction of Protein–Protein Interaction Networks

Cortical DEGs from sleep-deprived murine models were used to create a network of encoded proteins via ‘The Search Tool for the Retrieval of Interacting Genes (STRING) [22]. The protein–protein interactions (PPI) in the network were predicted using a medium probabilistic confidence score >0.4 and mapped with Cystoscope [23]. The application of a reasonably moderate interaction threshold was ensued to amplify coverage of potential protein interactions without overappraisal of their precision. Proteins naïve to interactions were excluded from the network.

### 2.4. Identification of Regulatory Differentially Expressed Genes

The interactome of cortical DEGs from sleep-deprived murine models in the PPI network was analyzed using the overlap of 11 topological algorithms, namely, the following: Degree, Closeness, Betweenness, Radiality, Stress, EcCentricity, BottleNeck, Edge Percolated Component (EPC), Maximum Neighborhood Component (MNC), Density of Maximum Neighborhood Component (DMNC), and Maximal Clique Centrality (MCC) [24,25]. DEGs based on multi-algorithmic ranking at the top 3% of the network were regarded as regulatory genes in cortical sleep deprivation.

## 3. Results

### 3.1. Overview of Microarray Datasets 

The literature search of the GEO and PubMed databases yielded three expression datasets on the cerebral cortex of sleep-deprived mice (GSE6514 [26], GSE78215 [27], and GSE33491 [28]; Table S1). The former datasets included cortical tissue biopsies from C57BL/6J mice (8–10 weeks old) that were sleep deprived for 6 h (*n* = 15) and from circadian and age-matched controls that were left undisturbed (*n* = 15).

### 3.2. Regulatory Differentially Expressed Genes in Sleep-Deprived Murine Cortex 

**Figure 1:**
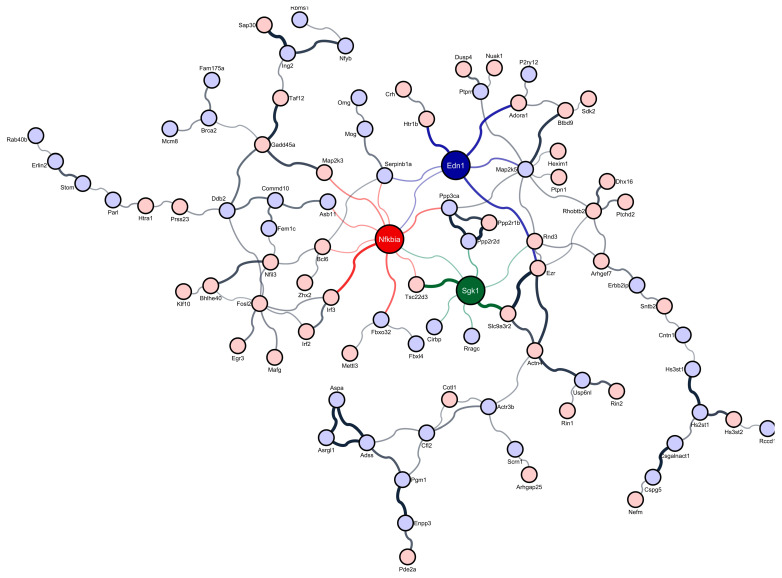
Regulatory genes (NFKB Inhibitor Alpha (NFKBIA), Ezrin (EZR) and Serum/Glucocorticoid Regulated Kinase 1 (SGK1)) in the protein–protein interaction network of cerebral cortex differentially expressed genes from murine models that were sleep deprived for 6 h and circadian-matched control that were left undisturbed. Red nodes indicate upregulated genes and blue nodes display downregulated genes.

A total of 195 cortical DEGs were retrieved in sleep-deprived murine models when compared to circadian controls (Table S2). Of these, 91 DEGs showed increased cortical expression and 104 DEGs exhibited decreased cortical expression. A network of a total of 89 encoded cortical proteins with 110 interactions of DEGs in response to sleep deprivation was constructed. Multi-algorithmic topological analysis of their interactome revealed the following three highest-ranked regulatory genes: NFKB Inhibitor Alpha (NFKBIA; *p* = 7.72 × 10^−3^, *Z* = 4.10), Ezrin (EZR; *p* = 6.34 × 10^−4^, *Z* = 4.87), and Serum/Glucocorticoid Regulated Kinase 1 (SGK1; *p* = 1.82 × 10^−2^, *Z* = 3.80) (Figure 1, Tables S3–S5).

## 4. Discussion

**Figure 2:**
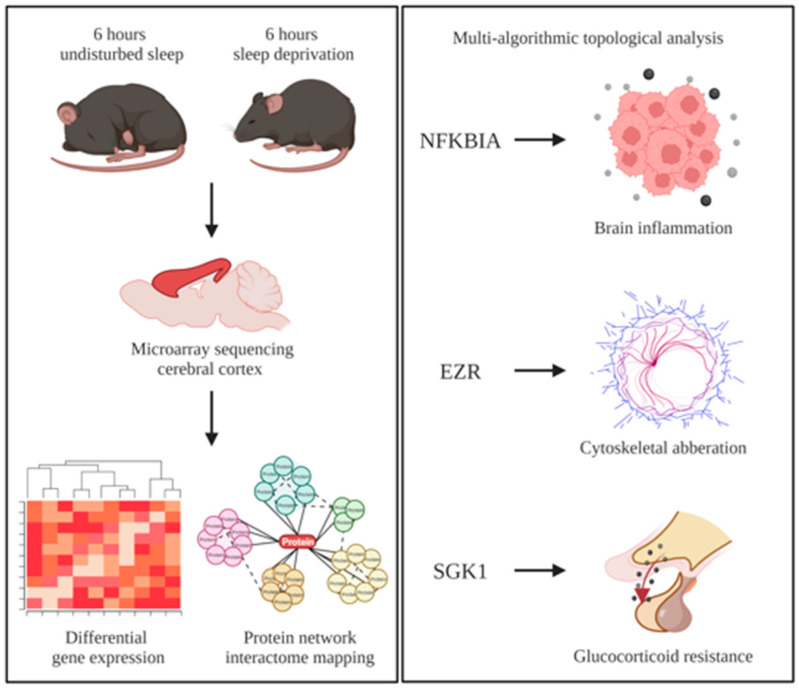
Cortical homeostasis in response to sleep deprivation is accompanied by the overexpression of three regulatory genes (NFKB Inhibitor Alpha (NFKBIA), Ezrin (EZR) and Serum/Glucocorticoid Regulated Kinase 1 (SGK1)), which are strong markers of increased brain inflammation, cytoskeletal aberrations, and glucocorticoid resistance and whose dysregulated expression changes imply aging-like transcriptional responses.

Multi-algorithmic topological analysis of DEGs in the cerebral cortex of sleep-deprived murine models, identified the following three regulatory genes: NFKBIA, EZR and SGK1. These genes may have a potential role as candidate markers of murine cortex homeostasis in response to sleep loss (Figure 2).

NFKBIA is responsible for the negative feedback regulation of NF-kappa B (NF-kB) activation that regulates the expression of genes involved in cell signaling of innate immunity [29]. A microarray analysis of the mouse central nervous system revealed that increased NFKBIA expression following melatonin treatment correlated with neuroprotective and anti-inflammatory activity [30]. Melatonin is an internal synchronizer of circadian rhythms. Melatonin is produced from the pineal gland located in in the posterior aspect of the cranial fossa [31], where its synthesis can be induced by macrophages [32]. Sleep deprivation may disrupt the immune-pineal axis via NF-kB activation and promote increases in circulating interleukin-6, tumor necrosis factor-alpha, and C-reactive protein [33]. Oxidative stress and systemic inflammation have also been associated with cortical thinning [34,35,36,37], specifically neuronal apoptosis in the prefrontal [38] and motor cortices [39]. Thus, dysregulation of NFKBIA, which may be partially determined by sleep deprivation and melatonin disruption, could trigger immune responses responsible for increased brain inflammation and accelerate aging [40].

SGK1 is a serine-threonine kinase that is regulated by glucocorticoid hormones, mediating cell survival, proliferation, differentiation, and apoptosis via phosphorylation of Mouse Double Minutes 2-dependent p53 degradation [41]. Chronically elevated circulating glucocorticoid levels may alter brain SGK1 expression in rat models, downregulating glucocorticoid receptor responsiveness [42], impairing neuronal plasticity [43], and oligodendrocyte morphology [44]. These changes may be explained, in part, by activation of NF-kB and subsequent expression of NMDA receptor NR2A and NR2B subunits [45]. Particularly, an increased concentration of glucocorticoids may disrupt the hypothalamic-pituitary-adrenal axis and promote aberrant glucocorticoid receptor activation [46]. Interestingly, previous research has demonstrated that SGK1 may upregulate glucocorticoid receptor phosphorylation, counteracting the cortisol-induced reduction in hippocampal neurogenesis from anxiety and depressive-like behaviors [47,48,49]. Indeed, decreased expression of SGK1 in the prefrontal cortex via traumatic stress may contribute to helplessness- and anhedonic-like behaviors in rodents [50]. Studies in sleep-deprived rodents have shown considerably increased response expression of SGK1 [51,52,53,54], with its expression further increased after long-term with respect to short-term SD [55,56,57,58]. During sleep deprivation, the prefrontal cortex interacts with the medial temporal lobe in memory encoding and recall stages [59], as well as serial subtraction [60], executive function [61], and attention processes [62,63]. Finally, SGK1 is involved in the under-expression of brain-derived neurotrophic factors and the vascular endothelial growth factor, leading to neuronal degeneration that is a consequence of aberrant glucocorticoid receptor activation [64,65]. Therefore, a close link between sleep deprivation and advancing brain aging may be described by dysregulated SGK1 expression that leads to an increased risk of cognitive deficits.

EZR is an astrocytic member of the ezrin-radixin-moesin protein family, involved in peripheral astrocyte process motility [66]. Specifically, EZR has a pivotal role in the connection of the intracellular actin cytoskeleton with transmembrane proteins, which are essential for cell signaling, development, proliferation, and migration [67,68]. Interestingly, EZR expression is mediated by radial glia and migrating cells of the cerebral cortex [69] and has been used as a marker of brain injury [70], cognitive impairment, and neurodegenerative disease [71]. During sleep deprivation, dysregulated EZR phosphorylation status has been linked with dry eyes and disrupted microvilli morphology of superficial corneal epithelial cells that is crucial for hydrating and protecting tear film adhesion from pathogenic infiltration [72]. Lastly, EZR activity is linked with the extracellular matrix and cytoskeleton through the Ras-like guanosine triphosphatase Gem, which drives sleep-wake-dependent elongation of peripheral astrocytic processes [73]. Taken together, EZR expression may explain cortical alterations and cytoskeletal abnormalities experienced during aging.

Although our findings make a novel contribution to the literature, an important consideration in the current study is the age of the mice, which was relatively young (8–10-week-old, young adult mice). Nevertheless, this work highlights possible age-dependent changes in gene expression with sleep deprivation. Understanding the unique neurophysiological underpinnings of cognitive impairments in young vs. aged animals is critical given that sleep impairments throughout life serve as a risk factor for neurodegenerative states in older age [74,75]. Indeed, previous work on proteomics shows the independent effects of sleep deprivation on young vs. older brains. For example, sleep deprivation differentially alters the expression of multiple proteins associated with neuroplasticity in young (2.5 months) vs. aged (24 months) mouse cortices [76]. Sleep deprivation also produces opposite effects on synaptic proteins in very young (3 weeks) and young adult (8 weeks) mice. Future work should focus on continuing to understand the unique developmental neurophysiological fingerprints of sleep impairments. Prospective outlooks of our analysis beyond the cerebral cortex using deep RNA sequencing will allow us to explore potential brain region-specific dimorphisms in gene responses following sleep deprivation.

## 5. Conclusions

Sleep deprivation can lead to brain deterioration, including cognitive decline and the onset of neurodegeneration, all of which advance with age. Sleep insufficiency and cognitive impairments are characterized by progressive neuronal losses in the cerebral cortex. Current evidence on cortical gene expression dynamics linked to sleep deprivation is scarce. Our study revealed three regulatory genes (NFKBIA, EZR, and SGK1) that exhibited the highest multi-algorithmic topological significance among DEGs in the cerebral cortex of sleep-deprived murine models. These genes constitute strong markers of increased brain inflammation, cytoskeletal abnormalities, and glucocorticoid resistance. Therefore, gene expression changes underpinning murine cortex homeostasis during sleep deprivation imply aging-like transcriptional responses. Future experimental human studies are warranted to validate the functional associations of these genes in sleep deprivation, across sleep-wake states, and vis-à -vis gene expression from the aging cortex. 

## Data Availability

The datasets generated and/or analyzed during the current study are available in the Gene Expression Omnibus repository, under the following accession numbers: GSE6514, GSE78215, and GSE33491.

## Supplementary Materials

The following supporting information can be downloaded at: https://www.mdpi.com/article/10.3390/brainsci12070825/s1, Table S1. Characteristics of the gene expression datasets included in the analysis, Table S2. Differentially expressed genes of the cerebral cortex between mice that were sleep deprived for 6 h and circadian-matched controls that were left undisturbed, Table S3. Interactome of the protein-protein interaction network of differentially expressed genes of the cerebral cortex between mice that were sleep deprived for 6 h and circadian-matched controls that were left undisturbed. Ranking was ensued based on the intersection of 11 topological algorithms. Percolated Component (EPC), Maximum Neighborhood Component (MNC), Density of Maximum Neighborhood Component (DMNC), Maximal Clique Centrality (MCC), Table S4. Highest-interacting genes in the protein-protein interaction network of differentially expressed genes of the cerebral cortex between mice that were sleep deprived for 6 h and circadian-matched controls that were left undisturbed, Table S5. Characteristics of highest-interacting genes in the protein-protein interaction network of differentially expressed genes of the cerebral cortex between mice that were sleep deprived for 6 h and circadian-matched controls that were left undisturbed.
