# Supplementary material for: Gene Expression Changes of Murine Cortex Homeostasis in Response to Sleep Deprivation Hint Dysregulated Aging-like Transcriptional Responses
Source: Brain Sci. 2022 Jun 24;12(7):825. doi: 10.3390/brainsci12070825 (PMC9313387; doi:10.3390/brainsci12070825)
Supplement: Supplementary file 1 [file brainsci-12-00825-s001.zip › brainsci-1746501-supplementary.pdf]

Table S1. Characteristics of the gene expression datasets included in the analysis.

Characteristics	Gene expression datasets		
	**GSE6514**	**GSE78215**	**GSE33491**
Sex	Male	Male	Male
Strain model	C57BL/6J	C57BL/6J	C57BL/6J
Control samples (*n*, age)	*n* = 5, 10-weeks old (±1 week)	*n* = 7, 2-months old	*n* = 3, 10-weeks old
Test samples (*n*, age)	*n* = 5, 10-weeks old (±1 week)	*n* = 7, 2-months old	*n* = 3, 10-weeks old
Cortical samples	M1 and M2 regions	Whole left cortex	Whole cortex
Experimental conditions	– SD was performed through gentle handling<br>– SD started at ZT0 (7:00 AM) and fished at ZT6 (13:00 AM)<br>– Tissue was collected immediately afterwards	– SD was performed through brushing<br>– SD started at ZT0 (7:30 AM) and fished at ZT6 (13:30 AM)<br>– Started at ZT0 and finished ZT6<br>– Tissue was collected immediately afterwards	– NA<br>– NA<br>– Tissue was collected immediately afterwards
Microarray platform	GPL1261	GPL17400	GPL6096

Table S2. Differentially expressed genes of the cerebral cortex between mice that were sleep deprived for 6 hours and circadian-matched controls that were left undisturbed.

**Gene ID**	***P*-value**	***Z*-score**	**Gene name**
Upregulated			
ACTN4	5.12E-03	4.27	actinin alpha 4
ADORA1	4.69E-02	3.40	adenosine A1 receptor
ARHGAP25	3.46E-02	3.53	Rho GTPase activating protein 25
ARHGEF7	1.55E-04	5.24	Rho guanine nucleotide exchange factor 7
ARID5A	3.95E-02	3.48	AT-rich interaction domain 5A
BCL6	1.32E-02	3.92	BCL6 transcription repressor
BHLHE40	7.70E-04	4.79	basic helix-loop-helix family member e40
BTBD9	1.56E-03	4.60	BTB domain containing 9
CALHM2	6.07E-03	4.20	calcium homeostasis modulator family member 2
CHRD	2.94E-03	4.43	chordin
CLDN2	3.04E-02	3.60	claudin 2
COTL1	4.73E-02	3.39	coactosin like F-actin binding protein 1
CRELD2	4.08E-06	6.29	cysteine rich with EGF like domains 2
CRH	1.17E-03	4.68	corticotropin releasing hormone
CSDC2	7.72E-03	4.10	cold shock domain containing C2
CTLA2A	4.92E-04	4.94	cytotoxic T lymphocyte-associated protein 2 alpha
DHX16	3.81E-02	3.49	DEAH-box helicase 16
DUSP4	9.42E-05	5.47	dual specificity phosphatase 4
EBP	3.03E-02	3.60	EBP cholestenol delta-isomerase
EGR3	7.33E-03	4.13	early growth response 3
EPHA10	3.41E-02	3.55	EPH receptor A10
EVC2	4.33E-02	3.44	EvC ciliary complex subunit 2
EZR	6.34E-04	4.87	ezrin
FAM20A	1.18E-03	4.67	FAM20A golgi associated secretory pathway pseudokinase
FAM53B	3.38E-03	4.38	family with sequence similarity 53 member B
Fam84a	6.69E-04	4.82	family with sequence similarity 84, member A
FEZF2	2.86E-02	3.63	FEZ family zinc finger 2
FOSL2	1.16E-05	5.86	FOS like 2, AP-1 transcription factor subunit
GADD45A	3.46E-04	5.04	growth arrest and DNA damage inducible alpha

HEXIM1	2.71E-04	5.11	HEXIM P-TEFb complex subunit 1
HS3ST2	2.86E-02	3.63	heparan sulfate-glucosamine 3-sulfotransferase 2
HTR1B	2.86E-02	3.63	5-hydroxytryptamine receptor 1B
HTR5B	2.43E-03	4.48	5-hydroxytryptamine (serotonin) receptor 5B
HTRA1	3.91E-02	3.49	HtrA serine peptidase 1
IRF2	4.52E-04	4.97	interferon regulatory factor 2
IRF3	3.45E-02	3.54	interferon regulatory factor 3
ITPK1	4.95E-02	3.37	inositol-tetrakisphosphate 1-kinase
JAGN1	1.27E-02	3.94	jagunal homolog 1
KLF10	6.07E-03	4.19	Kruppel like factor 10
LRFN2	5.51E-03	4.24	leucine rich repeat and fibronectin type III domain containing 2
MAFG	4.30E-02	3.44	MAF bZIP transcription factor G
MAP2K3	1.97E-02	3.78	mitogen-activated protein kinase kinase 3
METRNL	4.14E-02	3.46	meteorin like, glial cell differentiation regulator
METTL3	2.06E-03	4.53	methyltransferase 3, N6-adenosine-methyltransferase complex catalytic subunit
MIDN	5.62E-04	4.90	midnolin
MUP5	4.73E-02	3.39	major urinary protein 5
NEFM	4.34E-02	3.43	neurofilament medium chain
NFIL3	6.26E-03	4.18	nuclear factor, interleukin 3 regulated
NFKBIA	7.72E-03	4.10	NFKB inhibitor alpha
NOSTRIN	7.44E-03	4.11	nitric oxide synthase trafficking
NUAK1	1.55E-04	5.27	NUAK family kinase 1
P4HA2	2.86E-02	3.63	prolyl 4-hydroxylase subunit alpha 2
PCDH17	4.85E-02	3.38	protocadherin 17
PCP2	4.85E-02	3.38	Purkinje cell protein 2
PDE2A	4.72E-02	3.40	phosphodiesterase 2A
POM121	3.15E-02	3.58	POM121 transmembrane nucleoporin
PPP2R1B	3.03E-02	3.60	protein phosphatase 2 scaffold subunit Abeta
PRSS23	3.33E-02	3.56	serine protease 23
PTCHD2	1.78E-02	3.82	Patched domain-containing protein 2
PTPN1	1.27E-02	3.94	protein tyrosine phosphatase non-receptor type 1
RGS17	2.13E-03	4.52	regulator of G protein signaling 17
RHOBTB2	2.81E-02	3.64	Rho related BTB domain containing 2
RIN1	6.69E-04	4.84	Ras and Rab interactor 1

RIN2	1.55E-02	3.88	Ras and Rab interactor 2
RND3	2.29E-02	3.72	Rho family GTPase 3
SAMD4B	1.25E-04	5.35	sterile alpha motif domain containing 4B
SAP30	2.19E-02	3.74	Sin3A associated protein 30
SBK1	5.62E-04	4.91	SH3 domain binding kinase 1
SDF2L1	4.84E-06	6.15	stromal cell derived factor 2 like 1
SDK2	4.53E-02	3.42	sidekick cell adhesion molecule 2
SGK1	1.82E-02	3.80	serum/glucocorticoid regulated kinase 1
SLC43A1	2.45E-02	3.69	solute carrier family 43 member 1
SLC9A3R2	3.09E-03	4.41	SLC9A3 regulator 2
SNRK	3.46E-02	3.53	SNF related kinase
SNTB2	4.52E-04	4.98	syntrophin beta 2
SOX11	3.46E-02	3.53	SRY-box transcription factor 11
SPRED3	1.55E-04	5.25	sprouty related EVH1 domain containing 3
STAC3	4.33E-02	3.44	SH3 and cysteine rich domain 3
STARD8	3.41E-02	3.55	StAR related lipid transfer domain containing 8
TAF12	2.29E-02	3.72	TATA-box binding protein associated factor 12
TCN2	5.65E-03	4.23	transcobalamin 2
TJAP1	2.65E-02	3.67	tight junction associated protein 1
TMEM178A	6.69E-04	4.83	transmembrane protein 178
TNFRSF12A	4.17E-02	3.45	TNF receptor superfamily member 12A
TSC22D3	7.04E-06	6.03	TSC22 domain family member 3
TTYH3	1.27E-02	3.94	tweety family member 3
WFDC1	2.86E-02	3.62	WAP four-disulfide core domain 1
ZBTB40	9.47E-05	5.45	zinc finger and BTB domain containing 40
ZHX2	7.42E-03	4.12	zinc fingers and homeoboxes 2
ZNRF1	1.25E-04	5.36	zinc and ring finger 1
1700123O20Rik	2.02E-02	3.77	RIKEN cDNA 1700123O20 gene
Downregulated			
A730017C20Rik	4.94E-03	-4.28	RIKEN cDNA A730017C20 gene
ACTR3B	3.35E-02	-3.55	actin related protein 3B
ADSS	3.03E-02	-3.60	adenylosuccinate synthetase, non muscle
ANKMY2	1.65E-02	-3.85	ankyrin repeat and MYND domain containing 2
APPL2	4.36E-02	-3.43	adaptor protein, phosphotyrosine interacting with PH domain and leucine zipper 2

ASB11	1.71E-02	-3.83	ankyrin repeat and SOCS box containing 11
ASPA	7.80E-03	-4.09	aspartoacylase
ASRGL1	3.76E-02	-3.50	asparaginase and isoaspartyl peptidase 1
ATPAF1	1.62E-02	-3.85	ATP synthase mitochondrial F1 complex assembly factor 1
BRCA2	1.44E-02	-3.90	BRCA2 DNA repair associated
CBR4	8.33E-03	-4.07	carbonyl reductase 4
CC2D2A	3.46E-02	-3.53	coiled-coil and C2 domain containing 2A
CCNDBP1	7.44E-03	-4.11	cyclin D1 binding protein 1
CDC42SE2	2.43E-03	-4.48	CDC42 small effector 2
CFL2	3.20E-02	-3.57	cofilin 2
CIRBP	2.71E-04	-5.12	cold inducible RNA binding protein
CNTLN	5.12E-03	-4.27	centlein
CNTN1	1.27E-02	-3.94	contactin 1
COBLL1	2.80E-02	-3.65	cordon-bleu WH2 repeat protein like 1
COMMD10	1.90E-02	-3.79	COMM domain containing 10
CSGALNACT1	4.99E-02	-3.37	chondroitin sulfate N-acetylgalactosaminyltransferase 1
CSPG5	3.32E-02	-3.56	chondroitin sulfate proteoglycan 5
CTDSPL	1.62E-02	-3.86	CTD small phosphatase like
DARS2	2.19E-02	-3.74	aspartyl-tRNA synthetase 2, mitochondrial
DDB2	2.80E-02	-3.64	damage specific DNA binding protein 2
DIS3L2	4.73E-02	-3.39	DIS3 like 3'-5' exoribonuclease 2
DNAJC18	4.69E-02	-3.40	DnaJ heat shock protein family (Hsp40) member C18
EDN1	4.63E-03	-4.30	endothelin 1
EFHC2	4.85E-02	-3.38	EF-hand domain containing 2
ENPP3	1.15E-02	-3.98	ectonucleotide pyrophosphatase/phosphodiesterase 3
ENPP5	7.33E-03	-4.13	ectonucleotide pyrophosphatase/phosphodiesterase family member 5
ERBB2IP	1.27E-02	-3.93	ERBB2 interacting protein
ERLIN2	1.80E-02	-3.81	ER lipid raft associated 2
FAM171B	4.69E-02	-3.40	family with sequence similarity 171 member B
FAM175A	2.64E-02	-3.67	Abraxas 1, BRCA1 A complex subunit
FAP	1.62E-02	-3.86	fibroblast activation protein alpha
FBXL21	4.17E-02	-3.45	F-box and leucine-rich repeat protein 21
FBXL4	6.51E-04	-4.85	F-box and leucine rich repeat protein 4
FBXO32	1.73E-02	-3.83	F-box protein 32

FEM1C	2.70E-02	-3.66	fem-1 homolog C
FIG4	3.45E-02	-3.54	FIG4 phosphoinositide 5-phosphatase
GAS5	3.32E-02	-3.56	growth arrest specific 5
GPR37	9.36E-04	-4.73	G protein-coupled receptor 37
HADH	9.36E-04	-4.73	hydroxyacyl-CoA dehydrogenase
HEXDC	1.27E-02	-3.95	hexosaminidase (glycosyl hydrolase family 20, catalytic domain) containing
HOOK2	6.07E-03	-4.20	hook microtubule tethering protein 2
HS2ST1	6.75E-03	-4.16	heparan sulfate 2-O-sulfotransferase 1
HS3ST1	2.70E-02	-3.66	heparan sulfate-glucosamine 3-sulfotransferase 1
ING2	3.45E-02	-3.54	inhibitor of growth family member 2
ITM2A	9.29E-04	-4.74	integral membrane protein 2A
KLF11	1.62E-02	-3.86	Kruppel like factor 11
KLHL23	5.94E-03	-4.21	kelch like family member 23
LACTB2	4.85E-02	-3.38	lactamase beta 2
LAMA2	3.00E-02	-3.61	laminin subunit alpha 2
LSS	1.93E-02	-3.79	lanosterol synthase
MAP2K5	4.62E-02	-3.41	mitogen-activated protein kinase kinase 5
MCM8	1.32E-02	-3.92	minichromosome maintenance 8 homologous recombination repair factor
MOG	2.16E-02	-3.75	myelin oligodendrocyte glycoprotein
NFYB	3.20E-02	-3.57	nuclear transcription factor Y subunit beta
NICN1	3.15E-02	-3.58	nicolin 1, tubulin polyglutamylase complex subunit
OMG	1.55E-04	-5.25	oligodendrocyte myelin glycoprotein
P2RY12	4.16E-02	-3.46	purinergic receptor P2Y12
PARL	1.78E-02	-3.82	presenilin associated rhomboid like
PGM1	3.91E-02	-3.48	phosphoglucomutase 1
PKIA	3.95E-02	-3.48	cAMP-dependent protein kinase inhibitor alpha
PLA2G4E	1.16E-05	-5.89	phospholipase A2 group IVE
PPP2R2D	2.29E-02	-3.72	protein phosphatase 2 regulatory subunit Bdelta
PPP3CA	3.03E-02	-3.60	protein phosphatase 3 catalytic subunit alpha
PTBP2	3.46E-02	-3.53	polypyrimidine tract binding protein 2
PTPRR	2.86E-02	-3.62	protein tyrosine phosphatase receptor type R
RAB40B	2.06E-03	-4.54	RAB40B, member RAS oncogene family
RARS2	7.33E-03	-4.13	arginyl-tRNA synthetase 2, mitochondrial
RASGEF1B	3.40E-04	-5.06	RasGEF domain family member 1B

RBMS1	4.62E-02	-3.41	RNA binding motif single stranded interacting protein 1
RCCD1	2.29E-02	-3.72	RCC1 domain containing 1
RPAP2	3.81E-02	-3.50	RNA polymerase II associated protein 2
RRAGC	1.27E-02	-3.94	Ras related GTP binding C
SCRN1	4.69E-02	-3.40	secernin 1
SERPINB1A	9.72E-03	-4.03	serine (or cysteine) peptidase inhibitor, clade B, member 1a
SESTD1	3.09E-03	-4.41	SEC14 and spectrin domain containing 1
SGTB	3.09E-02	-3.59	small glutamine rich tetratricopeptide repeat co-chaperone beta
SH3BGRL2	3.46E-03	-4.37	SH3 domain binding glutamate rich protein like 2
SLC35F5	7.44E-03	-4.11	solute carrier family 35 member F5
SLITRK1	1.27E-02	-3.93	SLIT and NTRK like family member 1
SPATA7	1.00E-02	-4.02	spermatogenesis associated 7
SQLE	6.54E-03	-4.17	squalene epoxidase
STARD4	3.81E-02	-3.50	StAR related lipid transfer domain containing 4
STOM	4.24E-02	-3.45	stomatin
SYNM	4.69E-02	-3.40	synemin
SYT10	4.64E-02	-3.41	synaptotagmin 10
TCEA2	3.42E-03	-4.38	transcription elongation factor A2
TMEM132D	5.64E-03	-4.23	transmembrane protein 132D
TMEM87B	2.19E-02	-3.74	transmembrane protein 87B
TSPYL2	3.46E-02	-3.53	TSPY like 2
USP28	2.45E-02	-3.70	ubiquitin specific peptidase 28
USP6NL	2.43E-03	-4.48	USP6 N-terminal like
VPS4A	4.33E-02	-3.44	vacuolar protein sorting 4 homolog A
ZFP101	4.17E-02	-3.46	zinc finger protein 101
ZFP239	1.80E-02	-3.81	zinc finger protein 239
ZFYVE28	6.07E-03	-4.20	zinc finger FYVE-type containing 28
1700016K19Rik	1.26E-02	-3.96	RIKEN cDNA 1700016K19 gene
2410002F23Rik	2.80E-02	-3.65	RIKEN cDNA 2410002F23 gene
2610524H06Rik	2.29E-02	-3.72	RIKEN cDNA 2610524H06 gene
4930412C18Rik	2.48E-02	-3.69	RIKEN cDNA 4930412C18 gene

Table S3. Interactome of the protein-protein interaction network of differentially expressed genes of the cerebral cortex between mice that were sleep deprived for 6 hours and circadian-matched controls that were left undisturbed. Ranking was ensued based on the intersection of 11 topological algorithms. Percolated Component (EPC), Maximum Neighborhood Component (MNC), Density of Maximum Neighborhood Component (DMNC), Maximal Clique Centrality (MCC).

Gene ID	MCC	DMNC	MNC	Degree	EPC	BottleNeck	EcCentricity	Closeness	Radiality	Betweenness	Stress
NFKBIA	1	1	1	1	1	1	2	1	1	1	1
EZR	5	3	3	5	2	4	2	2	2	3	2
SGK1	3	3	3	3	4	2	1	3	3	4	6
EDN1	6	3	3	6	5	3	2	5	4	5	4
ACTN4	9	3	3	8	14	5	13	16	16	2	3
MAP2K5	2	25	25	2	3	10	2	4	6	9	5
PPP3CA	9	3	3	8	8	7	2	8	8	29	28
RND3	9	25	25	8	6	6	2	6	5	10	14
SERPINB1A	6	1	1	8	7	29	13	7	7	30	31
FOSL2	3	3	3	3	15	18	29	11	23	18	17
IRF3	19	3	3	19	17	18	13	10	12	19	18
BCL6	9	3	3	8	9	29	13	9	13	26	29
ACTR3B	9	3	3	8	36	7	29	29	36	7	8
SLC9A3R2	19	3	3	19	11	51	2	13	9	23	24
RHOBTB2	6	25	25	6	10	29	13	12	15	22	20
ARHGEF7	19	25	25	19	18	7	13	24	20	8	10
GADD45A	9	25	25	8	28	14	29	18	22	6	9
MAP2K3	34	25	25	34	22	10	13	14	9	12	12
DDB2	9	25	25	8	19	18	48	23	35	14	7
NFIL3	9	3	3	8	16	51	29	20	27	41	33
ASB11	34	25	25	34	21	10	13	17	14	31	25
PPP2R2D	19	3	3	19	13	51	2	21	17	53	53
COMMD10	19	25	25	19	25	14	29	25	26	32	27
CFL2	9	3	3	8	45	18	48	42	56	16	19
FBXO32	19	25	25	19	29	29	13	19	17	33	43
TSC22D3	34	3	3	34	12	51	2	15	9	55	55
ADORA1	19	25	25	19	20	40	13	22	19	42	40
PTPRR	19	25	25	19	30	29	13	28	31	33	34

BTBD9	19	25	25	19	23	40	13	27	30	43	38
ERBB2IP	34	25	25	34	43	10	29	46	40	11	13
BHLHE40	19	3	3	19	26	40	48	37	44	44	44
PPP2R1B	34	3	3	34	24	51	13	33	28	55	55
USP6NL	19	25	25	19	40	29	29	38	41	33	35
HTR1B	34	25	25	34	32	40	13	26	21	44	51
IRF2	34	3	3	34	27	51	29	30	29	55	55
ADSS	9	3	3	8	58	29	62	57	67	37	36
TAF12	34	25	25	34	48	25	48	41	44	24	32
BRCA2	19	25	25	19	51	29	48	39	48	33	45
MOG	34	25	25	34	39	40	29	34	32	44	48
PRSS23	34	25	25	34	46	23	62	49	57	21	11
SNTB2	34	25	25	34	63	14	48	62	59	13	15
PGM1	19	3	3	19	56	29	62	61	67	37	36
CIRBP	61	25	25	61	33	51	2	31	24	55	55
RRAGC	61	25	25	61	35	51	2	31	24	55	55
ING2	19	25	25	19	65	27	62	56	63	27	39
COTL1	34	3	3	34	49	51	48	53	58	55	55
FEM1C	34	25	25	34	31	51	48	40	47	54	54
CNTN1	34	25	25	34	71	17	62	70	70	15	21
HEXIM1	61	25	25	61	34	51	13	35	32	55	55
PTPN1	61	25	25	61	37	51	13	35	32	55	55
HTRA1	34	25	25	34	68	25	71	64	67	24	16
SCRN1	34	25	25	34	53	40	48	55	60	44	46
HS3ST1	34	25	25	34	77	18	71	73	74	16	22
HS2ST1	19	25	25	19	76	23	78	79	80	20	26
PTCHD2	61	25	25	61	38	51	29	44	38	55	55
ZHX2	61	25	25	61	41	51	29	43	37	55	55
DHX16	61	25	25	61	42	51	29	44	38	55	55
PARL	34	25	25	34	75	27	78	72	75	28	23
METTL3	61	25	25	61	54	51	29	50	42	55	55
P2RY12	61	25	25	61	50	51	29	52	46	55	55
FBXL4	61	25	25	61	59	51	29	50	42	55	55
EGR3	61	25	25	61	44	51	48	47	50	55	55

CRH	61	25	25	61	57	51	29	54	49	55	55
SDK2	61	25	25	61	52	51	29	58	52	55	55
ASPA	34	3	3	34	66	51	71	76	77	55	55
MAFG	61	25	25	61	47	51	48	47	50	55	55
ASRGL1	34	3	3	34	69	51	71	76	77	55	55
DUSP4	61	25	25	61	60	51	29	59	53	55	55
NUAK1	61	25	25	61	61	51	29	59	53	55	55
STOM	34	25	25	34	80	29	82	80	81	37	30
ENPP3	34	25	25	34	72	40	71	75	76	44	46
NFYB	34	25	25	34	78	40	71	71	72	44	52
CSGALNACT1	34	25	25	34	79	29	82	82	83	37	41
OMG	61	25	25	61	62	51	48	63	55	55	55
RIN1	61	25	25	61	64	51	48	66	61	55	55
RIN2	61	25	25	61	67	51	48	66	61	55	55
KLF10	61	25	25	61	55	51	62	65	64	55	55
HS3ST2	34	25	25	34	82	40	82	83	84	44	48
ERLIN2	34	25	25	34	83	40	85	85	85	44	42
CSPG5	34	25	25	34	84	40	85	86	86	44	48
FAM175A	61	25	25	61	70	51	62	68	65	55	55
MCM8	61	25	25	61	73	51	62	68	65	55	55
ARHGAP25	61	25	25	61	74	51	62	74	71	55	55
SAP30	61	25	25	61	81	51	71	78	73	55	55
RBMS1	61	25	25	61	86	51	78	81	79	55	55
PDE2A	61	25	25	61	85	51	78	84	82	55	55
RCCD1	61	25	25	61	87	51	85	87	87	55	55
RAB40B	61	25	25	61	88	51	88	88	88	55	55
NEFM	61	25	25	61	89	51	88	89	89	55	55

Table S4. Highest-interacting genes in the protein-protein interaction network of differentially expressed genes of the cerebral cortex between mice that were sleep deprived for 6 hours and circadian-matched controls that were left undisturbed.

Interactome rank	Gene name	Gene edges	Interacting genes
1	NFKBIA	8	ASB11 | BCL6 | FBXO32 | IRF3 | MAP2K3 | PPP3CA | SERPINB1A | TSC22D3
2	EZR	6	ADORA1 | EDN1 | HTR1B | MAP2K5 | NFKBIA | SERPINB1A
3	SGK1	7	CIRBP | NFKBIA | PPP2R2D | RND3 | RRAGC | SLC9A3R2 | TSC22D3

Table S5. Characteristics of highest-interacting genes in the protein-protein interaction network of differentially expressed genes of the cerebral cortex between mice that were sleep deprived for 6 hours and circadian-matched controls that were left undisturbed.

Gene ID	*P*-value	*Z*-score	Gene name
NFKBIA	7.72E-03	4.10	NFKB inhibitor alpha
EZR	6.34E-04	4.87	ezrin
SGK1	1.82E-02	3.80	serum/glucocorticoid regulated kinase 1
